# Rehabilitating homonymous visual field deficits: white matter markers of recovery—stage 2 registered report

**DOI:** 10.1093/braincomms/fcae323

**Published:** 2024-09-23

**Authors:** Hanna E Willis, Bradley Caron, Matthew R Cavanaugh, Lucy Starling, Sara Ajina, Franco Pestilli, Marco Tamietto, Krystel R Huxlin, Kate E Watkins, Holly Bridge

**Affiliations:** Wellcome Centre for Integrative Neuroimaging, FMRIB, Nuffield Department of Clinical Neuroscience, University of Oxford, Oxford OX3 9DU, UK; Department of Psychology, The University of Texas at Austin, Austin, TX 78712-1043, USA; Flaum Eye Institute and Center for Visual Science, University of Rochester, Rochester, NY 14642, USA; Wellcome Centre for Integrative Neuroimaging, FMRIB, Nuffield Department of Clinical Neuroscience, University of Oxford, Oxford OX3 9DU, UK; Wellcome Centre for Human Neuroimaging, Queen Square Institute of Neurology, UCL, Queen Square, London WC1N 3BG, UK; Department of Psychology, Department of Neuroscience, Center for Perceptual Systems, Center for Learning and Memory, The University of Texas at Austin, Austin, TX, USA; Department of Medical and Clinical Psychology, Tilburg University, Warandelaan 2, 5037 AB Tilburg, Netherlands; Department of Psychology, University of Torino, Torino 10123, Italy; Flaum Eye Institute and Center for Visual Science, University of Rochester, Rochester, NY 14642, USA; Wellcome Centre for Integrative Neuroimaging, Department of Experimental Psychology, University of Oxford, Oxford OX2 6GG, UK; Wellcome Centre for Integrative Neuroimaging, FMRIB, Nuffield Department of Clinical Neuroscience, University of Oxford, Oxford OX3 9DU, UK

**Keywords:** visual training, stroke, neuroimaging, diffusion-weighted imaging

## Abstract

Damage to the primary visual cortex or its afferent white matter tracts results in loss of vision in the contralateral visual field that can present as homonymous visual field deficits. Evidence suggests that visual training in the blind field can partially reverse blindness at trained locations. However, the efficacy of visual training is highly variable across participants, and the reasons for this are poorly understood. It is likely that variance in residual neural circuitry following the insult may underlie the variation among patients. Many stroke survivors with visual field deficits retain residual visual processing in their blind field despite a lack of awareness. Previous research indicates that intact structural and functional connections between the dorsal lateral geniculate nucleus and the human extrastriate visual motion-processing area hMT+ are necessary for blindsight to occur. We therefore hypothesized that changes in this white matter pathway may underlie improvements resulting from motion discrimination training.

Eighteen stroke survivors with long-standing, unilateral, homonymous field defects from retro-geniculate brain lesions completed 6 months of visual training at home. This involved performing daily sessions of a motion discrimination task, at two non-overlapping locations in the blind field, at least 5 days per week. Motion discrimination and integration thresholds, Humphrey perimetry and structural and diffusion-weighted MRI were collected pre- and post-training. Changes in fractional anisotropy (FA) were analysed in visual tracts connecting the ipsilesional dorsal lateral geniculate nucleus and hMT+, and the ipsilesional dorsal lateral geniculate nucleus and primary visual cortex. The (non-visual) tract connecting the ventral posterior lateral nucleus of the thalamus and the primary somatosensory cortex was analysed as a control. Changes in white matter integrity were correlated with improvements in motion discrimination and Humphrey perimetry. We found that the magnitude of behavioural improvement was not directly related to changes in FA in the pathway between the dorsal lateral geniculate nucleus and hMT+ or dorsal lateral geniculate nucleus and primary visual cortex. Baseline FA in either tract also failed to predict improvements in training. However, an exploratory analysis showed a significant increase in FA in the distal part of the tract connecting the dorsal lateral geniculate nucleus and hMT+, suggesting that 6 months of visual training in chronic, retro-geniculate strokes may enhance white matter microstructural integrity of residual geniculo-extrastriate pathways.

## Teaser

Willis *et al*. show that 6 months of motion discrimination training for stroke survivors with visual field deficits improved visual function. This improvement was accompanied by white matter microstructure changes in the distal section of the pathway between the dorsal lateral geniculate nucleus and visual motion area hMT+ post-training.

## Graphical Abstract

**Graphical Abstract:**
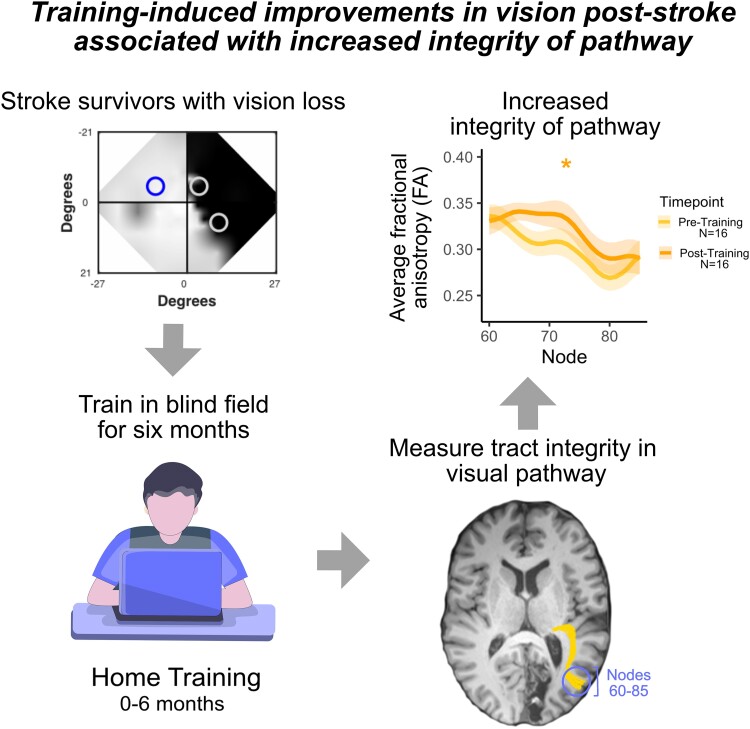

## Introduction

Retro-geniculate damage to the primary visual cortex (V1) or its immediate afferent tracts causes loss of conscious vision in contralateral portions of the visual field, referred to as homonymous visual field deficits (also know as hemi- or quadrantanopia). This type of vision loss affects between 20 and 57% of stroke survivors and significantly impacts activities of daily living, including mobility, reading and driving as well as quality of life.^1^ A brief 6-month period of spontaneous plasticity exists directly after stroke when visual deficits can improve.^2^ However, in contrast to those suffering from motor strokes,^3^ occipital stroke survivors are rarely provided with visual rehabilitation; when available, therapies that focus on compensatory eye movement strategies or substitution, such as prism lenses, are usually recommended.^4,5^ The reliance upon such therapies is controversial, with a recent Cochrane Review of randomized controlled trials concluding there was little evidence for the efficacy of current interventions.^1^

A more direct approach to improving vision in visual field deficits is to use training that repeatedly stimulates a portion of the blind field.^6,7^ These rehabilitation programmes require patients to discriminate visual stimuli within their blind field. They have successfully reduced the size of the visual deficit^8-12^ and improved contrast sensitivity,^11,13^ direction discrimination and orientation discrimination.^13-16^

Despite their success, the efficacy of visual training programmes remains highly variable across participants,^8-10,12,17^ and the reasons for this are poorly understood. We posit that variability in the extent and location of the stroke damage may help explain the inconsistency in visual improvement due to the specific fibres that are affected and their potential for plasticity to improve visual function.

Functional training changes the microstructure of white matter pathways and can be quantified using diffusion-weighted imaging. In stroke survivors, training increases fractional anisotropy (FA; a measure of myelination and organization of fibre tracts) in motor tracts, and this relates to motor improvement.^18^ FA is predictive of motor outcomes in skill training in healthy controls and stroke survivors.^19,20^ Based on this motor stroke literature, we predict that improvements in vision due to training in stroke survivors with visual field deficits should be associated with measurable changes in FA in white matter pathways connecting areas activated by training. There is currently no evidence to determine whether changes in FA can occur after visual restoration training in occipital stroke survivors.

While visual field deficits cause loss of normal, conscious vision, some patients retain the ability to detect or discriminate visual information in their blind field. This is known as ‘blindsight’ or residual vision, and has been shown to improve with practice.^21^ Studies suggest intact pathways between the ipsilesional lateral geniculate nucleus (dLGN) and extrastriate motion area (hMT+) are necessary for blindsight^22-25^. Based on these studies, we asked whether increases in FA in the entire dLGN-hMT+ pathway could underlie training-related improvements in motion discrimination thresholds in visual field deficits (Hypothesis 1). However, diffusion MRI tractography has previously shown significant overlap between dLGN-V1 and dLGN-hMT+ streamlines, and retrograde degeneration is known to affect the optic radiation that projects from dLGN to V1 after damage to the occipital lobe.^26,27^ A previous study therefore measured the distal, non-overlapping, portion of the dLGN-hMT+ as a purer measure of the pathway to hMT+ (between 60 and 85% of the total tract length).^23^ In an exploratory analysis, we also investigated this correlation for the distal portion of the dLGN-hMT+ pathway.

Blindsight is most reliably elicited by large (>4°), moving stimuli (5–20 Hz; ^13,28,29^). However, evidence suggests that vision beyond blindsight ability, such as global direction discrimination and integration^8,13,16^; and luminance detection—as in Humphrey perimetry—^9^ can also be improved by visual training in visual field deficits. Receptive fields in V1 are driven by small spots of light that vary in luminance,^30,31^ such as those used in Humphrey perimetry. Thus, training-induced improvements beyond blindsight abilities may result from bringing functionally impaired, spared V1 back ‘online’.^32,33^ We therefore additionally hypothesized that increased FA in the dLGN-V1 pathway may underlie training-related improvements in luminance detection (as measured by Humphrey perimetry; Hypothesis 2). Finally, we ascertained whether baseline measures of FA in the ipsilesional dLGN-hMT+ pathway can predict visual improvements induced by 6 months of training (Hypothesis 3). The protocol and planned analyses for this study were accepted by Brain Communications as a Stage 1 Registered Report in 2020.^34^ The study was also registered on clinicaltrials.gov (NCT04878861).

## Materials and methods

### Ethics and participants

#### Participants

Twenty-four stroke survivors were initially recruited (median = 49 years, range = 24–74 years, 6 female; see supplementary materials for power analysis) and provided written informed consent. Participants were healthy, MRI-safe, English-speaking adults with damage to V1 sustained in adulthood (18 years+) and causing homonymous visual field defects. They suffered damage at least 6 months prior to the study (i.e. in the chronic post-stroke phase, Supplementary Table 1). Twenty of these participants completed the rehabilitation training (see Supplementary Fig. 1 for individual visual fields). Four participants (R001, R008, R009 and R012; see Supplementary Fig. 2 for individual visual fields) were unable to complete the training protocol within the timeframe and therefore these data were removed from analyses exploring the rehabilitation. Two participants were unable to complete the MRI so were also removed from this analysis, leaving 18 who underwent MRI before and after training.

Finally, a subset of the participants (R001, R002, R003, R004, R005, R008, R010, R012 and R015) took part in a prior MRI study conducted between 2017 and 2018; their data were used for control analyses to assess if FA remained stable in the absence of training. Participants did not undergo any other visual rehabilitation therapy for the duration of the study. Phone interviews were conducted to determine eligibility and medical notes were also provided for this assessment.

#### Ethics

Ethical approval was given by the local ethics committee for the rehabilitation study (R60132/RE001) and for the pre-baseline MRI study (R59810/RE001). Before taking part, participants read the study information sheet and were made aware that the training to be administered was to inform research and was not an established treatment, nor could it be claimed to guarantee an improvement in vision. All participants provided informed consent and experiments were conducted in accordance with the ethical guidelines of the Declaration of Helsinki.

#### Exclusion criteria

Participants had no history of diagnosed cognitive or psychiatric disorders, including executive or attentional deficits. In addition, recruited participants had no history of eye disease or impairment other than visual field deficits, including all forms of visuospatial neglect. Participants were excluded from the analysis if they: (i) were unable to complete the required number of training sessions (minimum 100) within ∼6 months; (ii) were unable to complete the full testing protocol (all pre- and post-training behaviour and diffusion measures); (iii) were unable to fixate during training (see details below); (iv) showed fixation losses, false positives and negative errors outside of the normal range (≥20%) in either eye at any timepoint on the Humphrey Visual Fields (HVF).

### Study design

**Figure 1:**
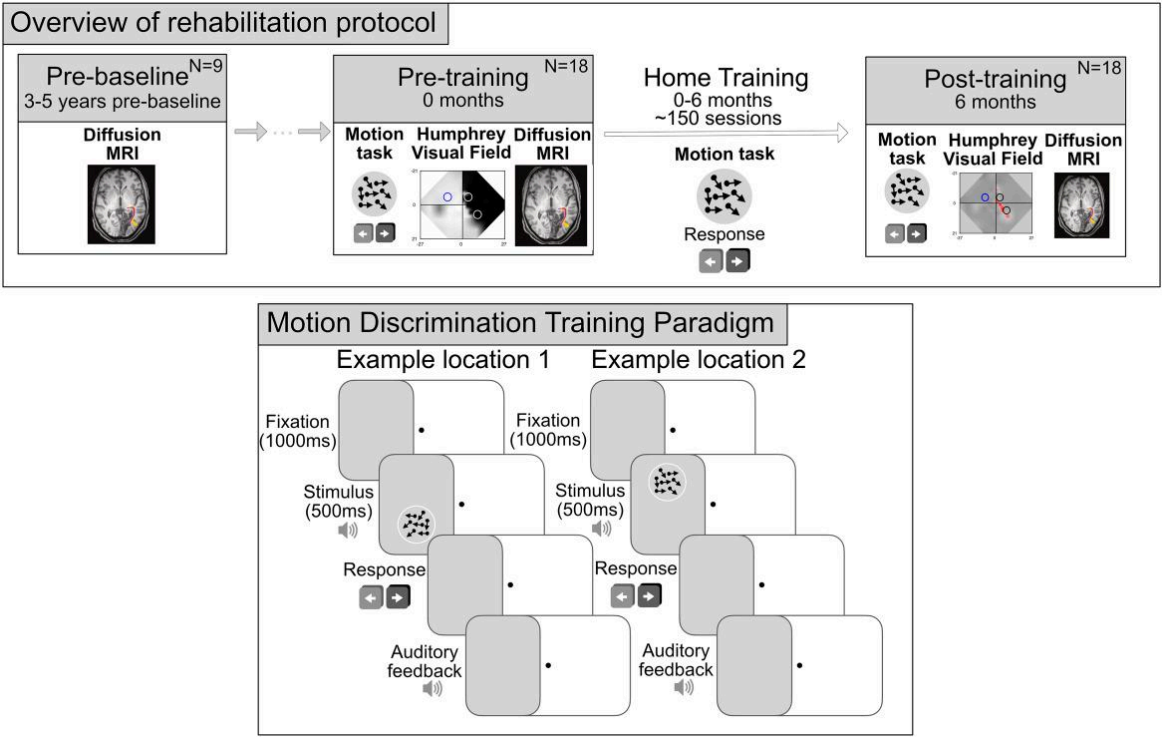
**Overview of rehabilitation protocol**. Study design indicating 2–3 research centre visits (upper). Pre-baseline (visit 1) involved a subset of participants who underwent MRI 3–5 years before the pre-training visit (visit 2). Visit 2 involved pre-training psychophysics, Humphrey perimetry and MRI, while the post-training visit (visit 3) involved post-training psychophysics, Humphrey perimetry and MRI. Participants performed 6 months of motion discrimination training carried out at home between visits 2 and 3. Motion discrimination home-training task was performed at two different locations in the blind field daily, while fixating on a centrally presented target (lower). Stimulus presentation was accompanied by a sound. Participants were asked to discriminate the global direction of movement (leftward or rightward) of the dots in the stimulus after each presentation. They were provided with auditory feedback signalling the correctness of their responses on each trial. This was repeated for 300 trials at each blind-field location, with each location trained independently in a block design. Number of participants (*N*) who completed each research visit is indicated.

Each participant visited the research centre for assessments on two–three occasions: pre-baseline (visit 1), pre-training (visit 2) and post-training (∼6 months later; visit 3; see Supplementary Table 1 for details regarding visits for each participant). The pre-baseline visit involved a subset of nine participants who also attended an additional research visit 3–5 years before the baseline visit as part of another study. All participants then completed at-home training between visits 2 (pre-training) and visit 3 (post-training). At-home training involved two sessions of visual training per day (∼40 min total), at least 5 days per week for 6 months. On average, participants completed 152.5 sessions (SD = 39.27; range = 102–271) over a 6-month period, and all participants completed at least 100 sessions (see Fig. 1 for schematic).

### Home-training paradigm

Participants were trained on a psychophysical, visual training programme at two locations in their blind field using stimulus and task parameters identical to those in prior studies.^8,13,16^ The training programme was designed in MATLAB (MathWorks) using the Psychophysics Toolbox.^35,36^ Participants used lab-issued chin-forehead rests and software customized to their own computer and monitor specifications (dimensions, resolution, refresh rate). The programme was compiled in MATLAB using the Compiler app (MathWorks) and sent to the participant. At visit 2, the research team assisted with set up of the programme and ensured participants were taught to use it appropriately. Participants were given instructions detailing how to set the training up at home to ensure consistency. They were encouraged to train when they were most awake, and the exact time was recorded in the daily logs produced by the training programme. Before training began, the programme presented a calibration square of a known size and participants were asked to measure the box size to ensure the screen dimensions were accurate. Participants completed training at one location and then the other sequentially. Results for each location were therefore analysed separately.

Participants were asked to make coarse, left-right discriminations of the global motion direction of random dot stimuli. Random dot stimuli consisted of black dots on a grey background (dot speed, 10°; dot lifetime, 250 ms; stimulus duration, 500 ms; aperture, 5° diameter). The participant’s eyes were positioned 42 cm away from the computer screen with a chin-forehead rest. Training difficulty was modulated using a 3:1 staircase: after three correct responses, the direction range increased from 0 to 360° in 40° steps, and after one incorrect response, it decreased by 40°. Auditory feedback signalled correct and incorrect responses on each trial. Performance for each session was calculated as a function of direction range level (see Fig. 1 for task details).

A Weibull function was then fitted to the data with a criterion threshold of 72% correct. The session threshold was normalized to the maximum range of dot directions (360°) to generate a normalized direction range (NDR) using the following equation:


$$\begin{array}{rl} & \mathrm{NDR}\;\mathrm{threshold}\;(\text{\%})=(360^{\circ }\text{–}\mathrm{Weibull}\text{-}\mathrm{fitted}\\ & \;\mathrm{direction}\;\mathrm{range}\;\mathrm{threshold})/360^{\circ }\times 100 \end{array}$$

Initial training locations were selected by measuring NDR thresholds sequentially, starting with the edge of the stimulus at the vertical meridian, and moving 1° laterally until performance dropped to chance (50% correct) and NDR thresholds were unmeasurable (designated as 100%). For all training locations, the full diameter of the stimulus was inside the Humphrey-defined blind-field border (see supplementary materials for training locations in all participants). During at-home training, as performance at each location reached 72% correct and NDR thresholds stabilized over 5–10 consecutive sessions (less than 30% coefficient of variation), this training location was moved to a new location 1° further into the blind field, along the *x*-axis (Cartesian coordinate space), and daily training started anew.

After each at-home training session was completed, the software automatically generated a log file detailing trial-by-trial performance. These log files were emailed to the laboratory weekly by participants, allowing researchers to compute thresholds, and follow their progress.

Participants were asked to fixate centrally throughout home training. They were reminded that fixation was essential to ensure the visual target was presented at the intended training locations in their blind field. Prior experience with this approach has shown that participants are highly motivated and compliant.^8,13^ An Eyelink 1000 Plus eye tracker (SR Research Limited, Ontario, Canada) was also used at both research centre visits to verify fixation and ensure fixation-contingent stimulus presentation. Only after at-home training results and threshold improvements were verified in-lab with controlled fixation, were specific participants classified as having experienced visual improvement at their training locations.

### Behavioural testing and MRI acquisition during pre- and post-training visits

#### Behavioural analyses

##### Motion discrimination and integration ability

At visits 2 and 3, per cent correct performance and NDR thresholds on the trained motion discrimination task were calculated at two locations in the blind field, and a matched sighted-field location. Performance at visit 2 (pre-training) was then subtracted from performance at visit 3 (post-training) to calculate change in per cent correct and NDR thresholds.

##### HVF acquisition and analysis

Monocular 24–2 and 10–2 visual fields were collected using a Humphrey Field Analyser (SITA fast and SITA standard, respectively) by the same two trained researchers, to estimate the deficit location and severity of each participant pre-training and post-training. Visual acuity was corrected to 20/20 and eye tracking was controlled to ensure central fixation during this task. This allowed us to obtain a measure of fixation stability throughout the study. Data from participants who showed fixation losses, false positives and negative errors outside of the normal range (≥20%) in either eye at any timepoint were removed from further analyses. As such, HVF data from two participants (R002 and R014) were removed due to fixation losses outside the normal range.

HVF data were analysed as described previously.^9,15^ The Humphrey software provides information about the deviation from the mean for each HVF testing location compared with an age-corrected normal population (pattern deviation, PD). Given the homonymous nature of visual field deficits in this patient population, composite binocular HVF were generated by first averaging monocular luminance detection thresholds (dB) for both eyes. Binocular 24–2 and 10–2 HVFs were then combined at overlapping regions. Difference maps were generated by subtracting the pre-training composite maps from the post-training composite maps. This quantified the area of HVF where the sensitivity improved by more than 6 dB relative to pre-training (area of improvement). The area of visual field impairment was defined as those points with an impaired binocular average sensitivity of below 10 dB.

#### MRI data acquisition and analyses

##### MRI acquisition

**Figure 2:**
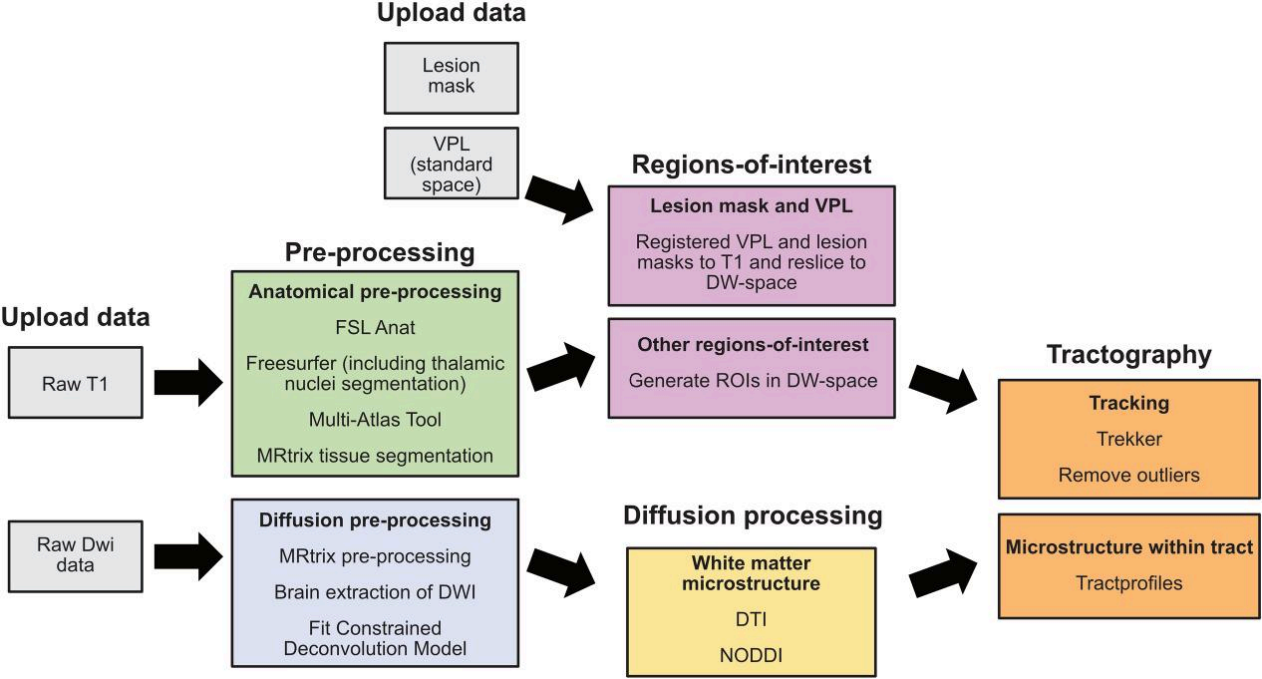
**Processing pipeline developed on brainlife.io.** Raw anatomy, diffusion-weighted (DW) data, lesion masks and ventral posterior lateral nucleus of the thalamus (VPL) masks in standard space (grey) were uploaded for each participant and timepoint. Anatomical data were pre-processed using apps listed (green), followed by the definition of regions of interest (pink). Lesion and VPL masks were registered to anatomical data and then resliced into DW-space. Additional regions of interest from the Glasser atlas were generated and resliced into DW-space. Diffusion data were pre-processed using apps listed (blue). Once data were pre-processed, DTI and NODDI models were fit to the data to quantify white matter microstructure. Tractography was then run between dLGN-V1, dLGN-hMT+ and VPL-S1 using the Trekker app. Tract outliers were removed using the remove outliers app. Finally, Tractprofiles was used to calculate white matter microstructure along the tract.

Scanning used a 3T Siemens Prisma MRI scanner with a 64-channel head coil at the research centre. A structural (T1w) scan was first acquired for each participant for registration and quantification of the lesion size. These were high resolution (1 × 1 × 1 mm^3^) whole head T1-weighted anatomical images (TE = 3.97 ms, TR = 1900ms, FoV = 192 mm, flip angle = 8°). Diffusion-weighted data were acquired using the UK Biobank sequence.^37^ We used a spin-echo echo-planar imaging sequence (EPI; TR = 3600 ms, TE = 92 ms, 2 × 2 × 2) in the anterior-posterior encoding direction with 50 *b* = 1000 s/mm^2^ and 50 *b* = 2000s/mm^2^ diffusion-weighted volumes acquired with 100 distinct diffusion-encoding directions and 3× multiband acquisition. Five volumes without diffusion weighting (b0 volumes; *b*-value = 0 s/mm^2^) and three additional b0 volumes (*b*-value = 0 s/mm^2^) with posterior-anterior encoding were also acquired to correct for image distortion. All MRI data acquired for this project were managed using the reproducible neuroscience platform *brainlife.io*.^38^ Initially, data were securely stored in a private project on *brainlife.io* and processed using state-of-the-art reproducible Apps^38^ (see Fig. 2 for diagram of processing pipeline). All data products were generated by tracking their provenance (i.e. keeping a record of the combination of the generated data product and processing application used to generate the data;^39^ see Supplementary Table 2 of all apps used).

##### Anatomical pre-processing

Raw anatomical (T1) images were pre-processed using the FSL tool *FSLanat* (brainlife.app.273). These images were cropped and reoriented to a standard MNI152 template using a linear transform. Cortical and white matter surfaces were generated using Freesurfer (brainlife.app.462). Following this, the Human Connectome Project multimodal parcellation (hcp-mmp) was mapped to the Freesurfer surfaces using the multi-Atlas Tool (brainlife.app.470). Pre-processed images were then segmented into tissue types (grey matter, white matter, CSF) using the MRTrix 5ttgen function (brainlife.app.239). The 5ttgen probability masks were used to generate a grey-white matter boundary for defining seeds and terminations for tractography (see Materials and methods: Tractography for more information regarding tractography procedures). Finally, thalamic subnuclei were segmented using Freesurfer’s segment_thalamic_nuclei functionality (brainlife.app.222).

##### Tracts of interest

Using the *brainlife.io* software, we performed analyses of specific tracts using diffusion tractography. The main pathway that has been implicated in residual processing in visual field deficits is between dLGN and hMT+. We also investigated changes in FA due to training in the dLGN-V1 and the VPL-S1 pathways. The latter acted as a control tract, as it relays sensory (non-visual) information between the thalamus and cortex and is unlikely to change as a result of visual training.

##### Regions of interest

dLGN masks were generated from Freesurfer’s segment_thalamic_nuclei function (brainlife.app.222). Masks of V1, hMT+ and S1 were derived from the Glasser Atlas.^40^ All masks were resliced into diffusion space using AFNI’s 3dROIMaker function (brainlife.app.592). VPL was derived from the Oxford Thalamic Connectivity Probability Atlas and was uploaded to *brainlife.io*. Lesion masks were manually delineated for each participant by 2–4 trained researchers and uploaded to brainlife. Lesion and VPL masks were then registered to acpc-aligned anatomical images (brainlife.app.670) and resliced into diffusion space (brainlife.app.671). V1 in the damaged hemisphere was then masked by the lesion to ensure that only preserved brain tissue was included in the definition (brainlife.app.680). To ensure that ROIs were consistent across scans and timepoints, ROIs were transformed from standard space to structural space.

##### Diffusion pre-processing

Raw diffusion images were pre-processed using MRTrix3 (bl.app.68). Pre-processing steps included: (i) reorientation to standard neurological orientation (*fslreorient2std* tool, FSL; ^41^), (ii) gradient orientation check (*dwigradcheck* command in MRTrix3; ^42^) (iii) PCA denoising (*dwidenoise* command in MRTrix3; ^43^) (iv) Gibbs de-ringing (*mrdegibbs* command in MRTrix3; ^44^) (v) susceptibility-weighted distortion correction of the reverse phase encoding directions (AP and PA) using FSL’s *topup* function,^45,46^ (vi) Eddy-current and motion-correction using the *Eddy* tool in FSL,^47^ (vii) de-bias using ANT’s n4 functionality,^48^ (viii) registration of DWI to the anatomical image (*epi_reg* in FSL;^49,50^) and re-slicing to 1 mm isotropic voxels. The FSL Brain Extraction tool in Brainlife (brainlife/app-FSLBET;^51^) was used to create brain masks of the pre-processed, acpc-aligned diffusion images.

Following pre-processing, the diffusion tensor imaging (DTI) models (brainlife/app-fslDTIFIT;^52^) and neurite orientation dispersion and density imaging (NODDI) models (brainlife.app.365;^53,54^) were fit to the pre-processed data. The constrained spherical deconvolution model^55^ was also fit to the pre-processed data to guide tractography using functions from MRTrix3 (brainlife.app.238) across 4 spherical harmonic orders (lmax; 2, 4, 6 and 8).

##### Tractography

Diffusion-weighted MRI data were pre-processed using a series of *brainlife.io* Apps (see Supplementary Table 2). Some of these Apps implement methods from major software libraries such as FSL,^41,46^ FreeSurfer,^56^ MRTrix,^57^ VISTASOFT and AFQ,^58,59^ LiFE and Ensemble Tractography.^60,61^ Anatomically constrained tractography and Ensemble Tractography were used to track the visual white matter tracts of interest (e.g. brainlife.app.226 or brainlife.app.297).^58,62^

Tractography between dLGN-hMT+, dLGN-V1 and VPL-S1 was conducted using Trekker (brainlife.app.355).^63^ For the VPL-S1 and dLGN-V1 tracts, the minimum and maximum length of streamlines were set at 10 and 200 respectively and a single lmax was used (set at 8). Due to sparse fascicles when tracking the dLGN-hMT+ tract, the parameters were adjusted, setting the minimum and maximum length of streamlines at 50 and 150 respectively and ensemble tracking^64^ across 4 spherical harmonic orders (i.e. lmax) was used. Hemisphere exclusions were used for all tracts to ensure that they remained within the same hemisphere. Streamlines were only included if they touched both regions of interest and travelled within white matter in the same hemisphere. The total number of streamlines was constrained to 1000 per tract of interest. The optimum curvature radius threshold, step size and fibre orientation were determined by the Trekker algorithm^63^ based on the voxel resolution of the pre-processed diffusion images. Although specific curvature radius and step sizes were described in the pre-registered report, it was determined that allowing Trekker to determine optimal values for these parameters produced the best outputs.

An anatomically informed approach was used to identify core fascicles.^23,58-60,65,66^ Outlier fascicles were removed from analyses to produce a conservative tract estimate (brainlife.app.195). Outlier fascicles were defined as those located >2.6 SD away from the core of the tract or 2.8 SD longer than the mean tract length, using a Gaussian distribution to represent fascicle length and distance. If this calculation was not possible due to the small number of sparse fascicles (<10), then it was assumed that tracking was not possible between two areas of interest. Data were removed from the dLGN-V1 tract and from the dLGN-hMT+ tract for two participants (R004 and R006) due to difficulties with tracking between ROIs for at least one timepoint. No data were removed from the VPL-S1 tract. Tracts of interest were defined anatomically as tracts that pass between the two ROIs in the same hemisphere.

##### White matter microstructure

We used both DTI (DTI; brainlife.app.292) and NODDI (Neurite Orientation Dispersion and Density Imaging; brainlife.app.35;^54^) models to estimate the microstructural properties of the white matter tracts at each timepoint. We then combined the microstructural parameters estimated via either DTI (FA and MD) or NODDI (Neurite density index, NDI; orientation dispersion index, ODI; isotropic volume fraction, ISOVF) with the spatial information of each tract trajectory using the Track Analysis Profiles app in brainlife (brainlife.app.361;^59^). This gave us a microstructural profile of each parameter weighted by distance from the mean of the tract at each trajectory. Each tract was resampled to 100 nodes, distributed equally along the length of the tract.^59^ The first and last 15 nodes (1–15 and 85–100) were removed to limit grey matter contamination and partial volume effects. These clipped profiles, containing the central 70 nodes, were then used to calculate measures of mean tract microstructural parameters (see Supplementary Table 3 for average FA at each timepoint in each tract of interest). These tract profiles were used to determine the white matter microstructure parameter for each participant and timepoint along tracts between dLGN and hMT+, dLGN and V1 and VPL and S1. This was calculated along the core of the tracts to reduce partial volume artefacts.

One limitation of standard tract-based diffusion-weighted imaging analyses is that diffusion properties are usually averaged over the length of the tract, obscuring how they might vary along the length of the tract.^59^ In humans, there is significant overlap between the dLGN-V1 and dLGN-hMT+ tracts and it is conceivable that the significant retrograde degeneration of the optic radiation that projects from dLGN to V1 after damage to the occipital lobe^26,27^ might mask training-induced changes in the dLGN-hMT+ tract. The distal, non-overlapping, portion of the dLGN-hMT+ (60–85% of the total tract length) has been previously linked to residual vision after V1 damage.^23^ Findings from this previous study motivated us to perform an exploratory analysis of the distal portion of the dLGN-hMT+ tract, after it branches away from the geniculate-radiation bundle, in the present data set. To this end, diffusion measurements were quantified at 100 nodes along the tract, before being clipped to contain only nodes 60–85.

## Statistical analyses

All statistical analyses were carried out in R studio (R version 4.1.2). Change scores were calculated for the motion discrimination task and diffusion metrics (FA, MD, NDI, ODI and ISOVF) by subtracting the pre-training score from the post-training score. Linear regression analyses were then used to determine the relationship between improvements in training and tract measures using the *stats* package. Linear mixed-effects models were used to determine differences along the distal dLGN-hMT+ tract between timepoints using the *lme4* package. *Post hoc* tests were carried out using the *emmeans* package. Bonferroni-Holm adjusted *P*-values were calculated using the *stats* package.

## Results

### Behavioural changes

Although participants trained at two different blind-field locations, the location that improved most (‘most improved location’) was used as a measure of improvement capacity in all analyses. Across all participants, paired *t*-tests confirmed a significant increase in per cent correct between pre- and post-training visits (mean ± SD pre = 53.8 ± 3.9%, post = 72.6 ± 11.2%; *t* (1,17) = −6.9, *P* ≤ 0.001). Moreover, there was a significant reduction in NDR thresholds (mean ± SD pre = 100%±0% versus post: 57.3%±33.4%; *t* (1,17) = 5.42, *P* ≤ 0.001), indicating improvement at the trained task after 6 months of training.

**Figure 3:**
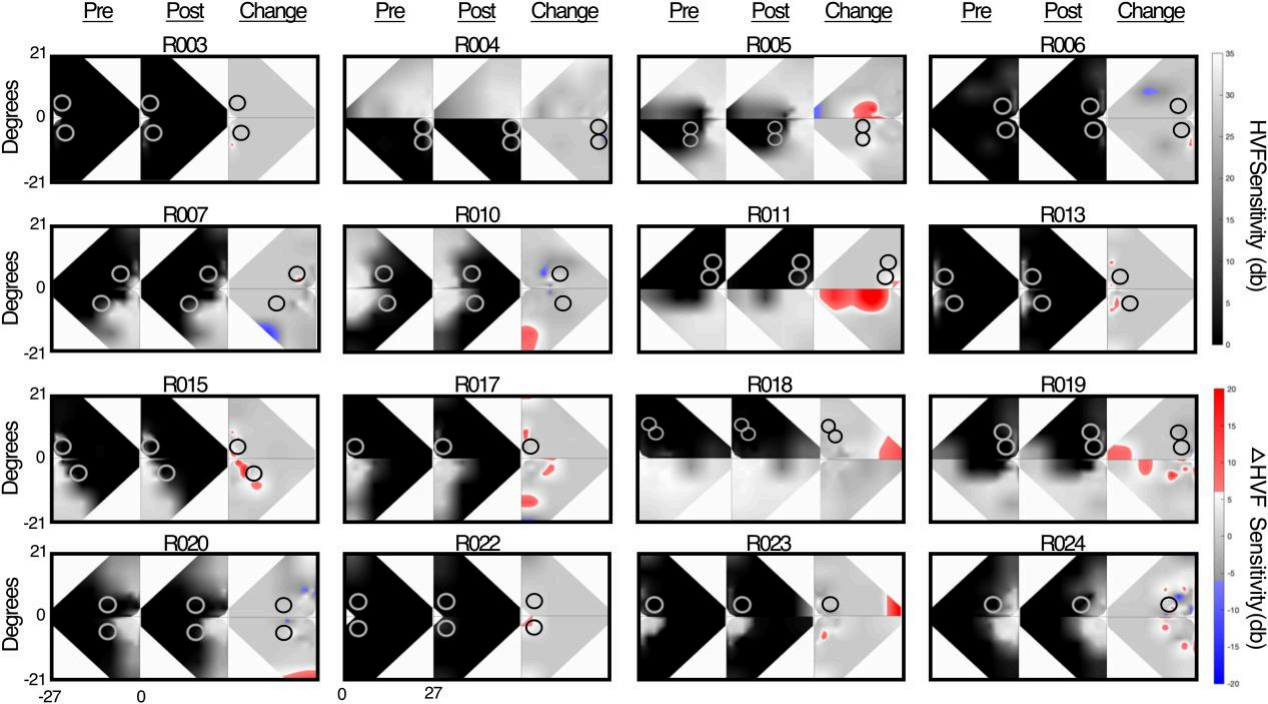
**Change in binocular composite Humphrey Visual Fields for all participants.** The pre-training visual field (left), post-training visual field (middle) and the change between the two (right). Areas highlighted in red are ‘areas of improvement’ by more than 6 decibels, while areas highlighted in blue are ‘areas of worsening’ by more than 6 decibels. The two training locations are indicated by circles. Participants R017, R023 and R024 only trained at one location. Participants R002 and R014 were removed from analyses due to reliability errors outside the normal range on one of the visual field tests.

There was also visual field improvement measured with Humphrey perimetry. Across participants, HVF sensitivity improved by >6 dB over a mean area 29.5 ± 35.8 deg^2^ in size (range: 0–134.9 deg^2^, red areas in Fig. 3). A one-sample *t*-test indicated that this change was significantly different from zero (*t*(1,15) = 3.5, *P* = 0.002). Thus, after 6 months of global motion discrimination training, there was an improvement in HVF perimetry, albeit with considerable variability between participants. In addition, a linear regression indicated that the magnitude of improvement on the motion discrimination task (NDR threshold) was predictive of the area of improvement on the HVF (adjusted *R*^2^ = 0.31, *F*(1,14) = 7.7; *P* = 0.015). Moreover, the area of improvement was not related to pre-training deficit size (adjusted *R*^2^=−0.03, *F*(1,18) = 0.5; *P* = 0.48).

### White matter parameters

#### Correlation between white matter changes and training-induced recovery

**Figure 4:**
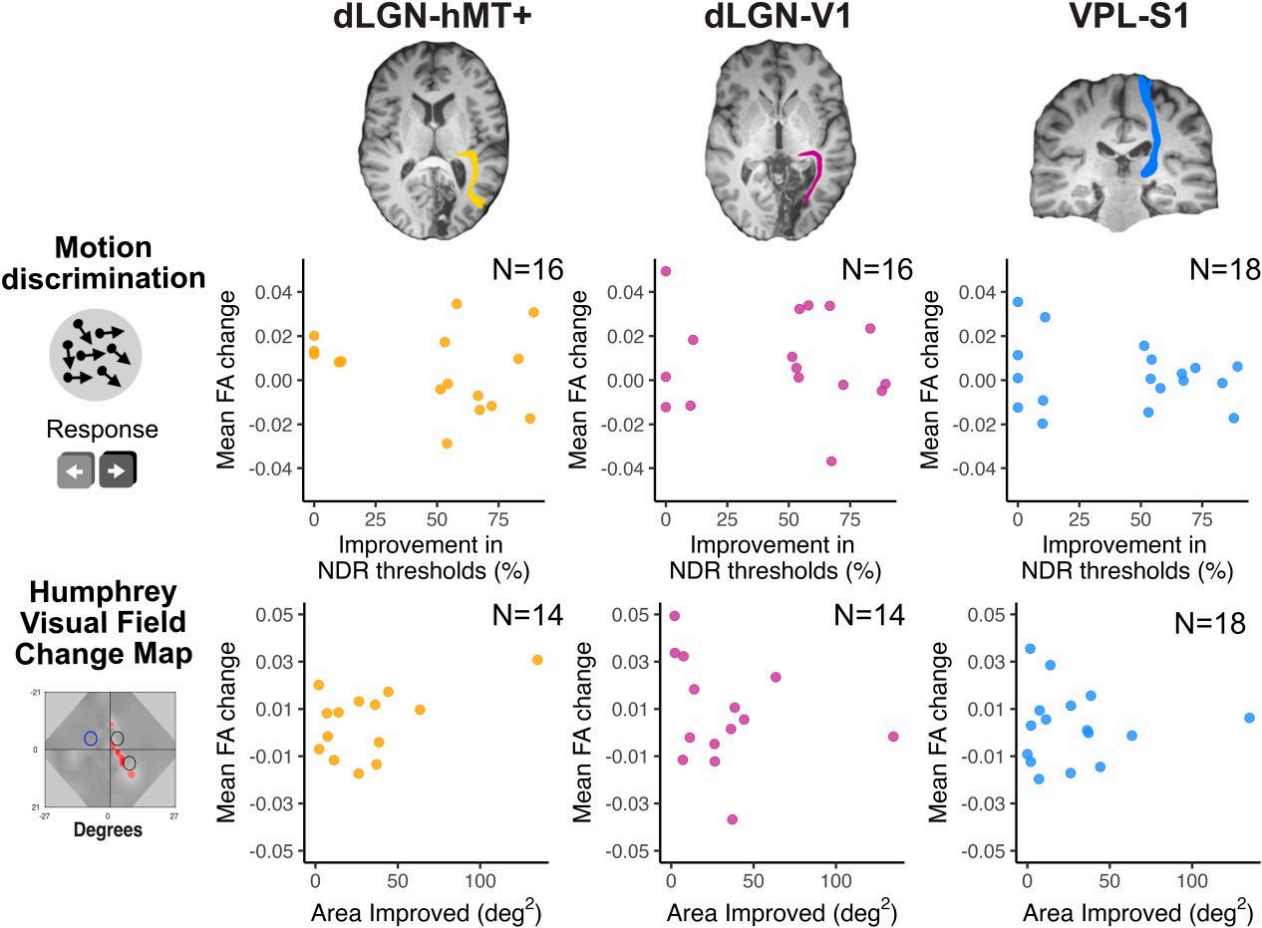
**Scatter plots showing lack of relationship between mean FA change in tracts of interest and behavioural change.** Plots show the relationship between FA in dLGN-hMT (left), dLGN-V1 (middle), VPL-S1 (right) and improvement in NDR on the trained motion discrimination task (upper) and area of improvement on the HVF (lower) between the pre- and post-training timepoints. There was no significant relationship between improvement on the trained task or area of improvement on the HVF and mean FA change across any of the tracts.

Linear regression analyses were used to determine the relationship between motion discrimination thresholds (hypothesis 1) and area of improvement on the HVFs (hypothesis 2) and changes in FA in the three tracts (ipsilesional dLGN-hMT+, dLGN-V1, VPL-S1). Contrary to our hypotheses, as can be seen in Fig. 4, there was no evidence of a significant relationship between (i) improvement in motion discrimination thresholds and increases in FA in the dLGN-hMT+ tract *(hypothesis 1; adjusted R^2^ = 0.004, F(1,14) = 1.1; P = 0.32)* or (ii) area of improvement on the HVF perimetry and increase in ipsilesional FA in the dLGN-V1 tract, after training *(hypothesis 2; adjusted R^2^=−0.01, F(1,12) = 0.1; P = 0.36)*. As expected, there was no relationship between change in FA in the ipsilesional VPL-S1 and improvements in motion discrimination thresholds due to training *(adjusted R^2^=−0.02, F(1,16) = 0.7; P = 0.41)* or area of improvement on the HVF *(adjusted R^2^=−0.07, F(1,14) = 0.0; P = 0.94).* Moreover, in an exploratory analysis, we also confirmed that improvement in motion discrimination thresholds was not related to increases in FA in the dLGN-V1 tract *(adjusted R^2^=−0.06, F(1,14) = 0.1; P = 0.74)* or to area of improvement on the HVF perimetry and increase in ipsilesional FA in the dLGN-hMT+ tract, after training *(adjusted R^2^ = 0.19, F(1,12) = 4.0; P = 0.07)*. R011 improved more than the other participants (134.86 deg^2^) and may be considered an outlier. When removed from the analysis there was still no relationship between FA in dLGN-V1 and area of improvement (*adjusted R^2^ = 0.02, F(1,11) = 1.2; P = 0.29)*.

#### Predictors of recovery

Linear regression was used to determine whether pre-training (visit 2) FA in the ipsilesional dLGN-hMT+ predicted improvement in motion discrimination thresholds. There was no evidence of a positive relationship between pre-training FA in the dLGN-hMT+ tract and improvement in motion discrimination thresholds (hypothesis 3; one-tailed; *adjusted R^2^ = 0.06, F(1,14) = 1.9; P = 0.19*). Similarly, no effect was found in dLGN-V1 (*adjusted R^2^=−0.06, F(1,14) = 0.1; P = 0.82)* or VPL-S1 tracts (adjusted *R*^2^=−0.04, *F*(1,16) = 0.3; *P* = 0.60).

### Exploratory analyses

#### Alternative white matter metrics

Due to our sample size and power calculation, we were only powered to detect large effect sizes across a small number of tests. We thus limited the number of hypotheses that are directional and registered to those based strongly on the literature. However, in addition to the planned analyses described above, we also explored alternative DTI (MD, RD, and AD) and NODDI (NDI, ODI, ISOVF) white matter metrics for the dLGN-hMT+ tract in relation to improvements in behaviour (motion discrimination and HVF). Linear regression analyses, Bonferroni-corrected for multiple comparisons i.e. *P* < 0.05/6 ≤ 0.008, were used to study the relationship between behaviour and these additional white matter metrics. No significant relationships were found between any diffusion measures and improvements in NDR thresholds or areas of improvement on the HVF (see Table 1).

**Table 1:** Linear regression analyses of change in normalized direction range threshold (NDR) or area of improvement on Humphrey Visual Fields (HVF) and additional diffusion metrics for the dLGN-hMT+ tract

Task	Diffusion measure	Adjusted *R*^2^	*F* Statistic	Degrees of freedom	*P* value
NDR threshold change	MD	−0.07	0.0	1,14	0.90
	RD	−0.05	0.3	1,14	0.62
	AD	−0.06	0.2	1,14	0.70
	NDI	−0.04	0.4	1,14	0.53
	ODI	0.01	1.1	1,14	0.31
	ISOVF	0.04	1.7	1,14	0.22
Area improved on HVF	MD	−0.08	0.0	1,12	0.99
	RD	−0.07	0.2	1,12	0.67
	AD	−0.06	0.3	1,12	0.60
	NDI	0.00	1.0	1,12	0.34
	ODI	−0.08	0.0	1,12	0.84
	ISOVF	0.07	2.1	1,12	0.18

#### White matter microstructure along the tract

**Figure 5:**
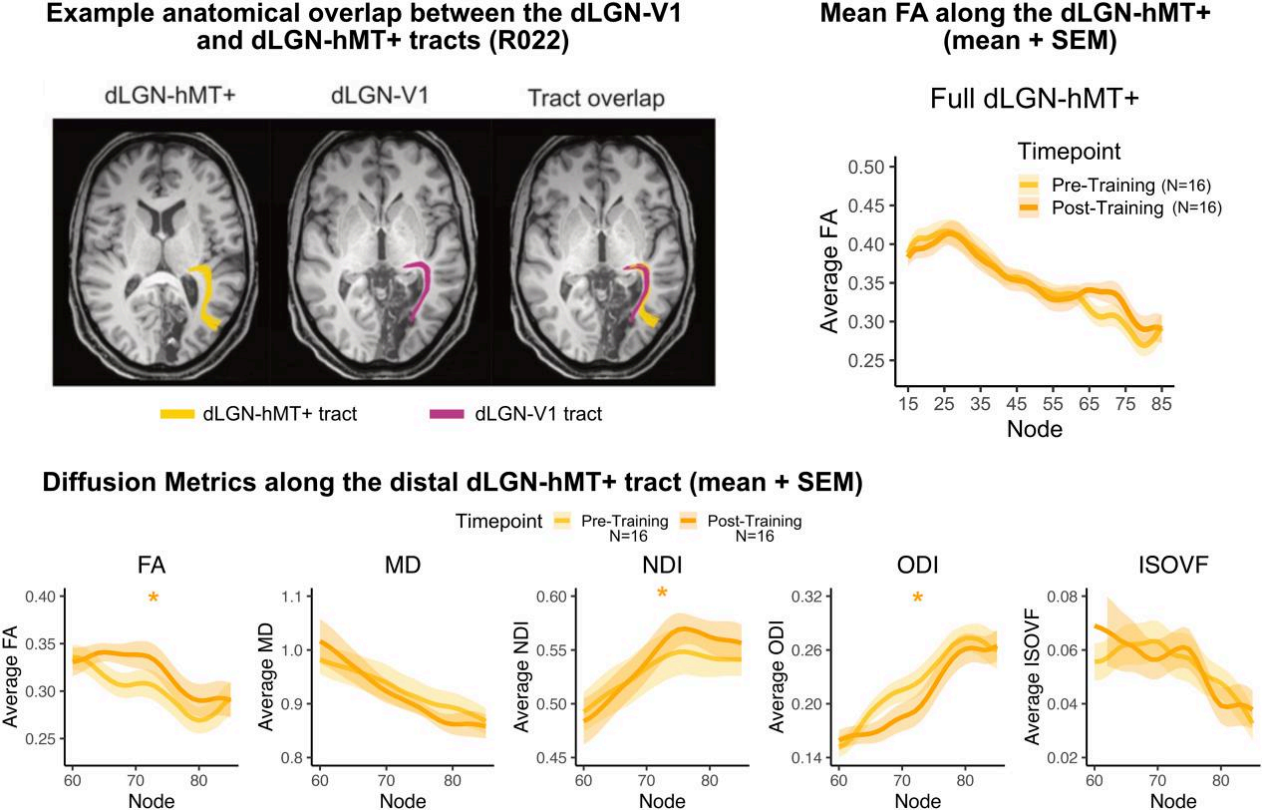
**The distal dLGN-hMT+ tract after training.** Upper left: Example overlapping visual tracts. Ipsilesional dLGN-hMT+ (left) and dLGN-V1 (middle) in one representative participant (R022) demonstrating the significant overlap (right). Upper right: Average mean group FA tract profile for the full dLGN-hMT+ tract for the Pre-Training and Post-Training timepoints. Lower: Diffusion metrics along the distal portion of the dLGN-hMT+ are shown. Lines represent the mean diffusion metric across participants at each node, while shaded error bars reflect the standard error of the mean (SEM). Significant differences between visits 2 and 3 (pre- to post-training) as measured by a linear mixed-effects model are indicated by an asterisk (*). There was a significant increase in FA (est = −0.019, SE = 0.003, *P* < 0.001) and neurite density index (NDI; est = 0.009, SE = 0.003, *P* = 0.001) and decrease in orientation dispersion index (ODI; est = −0.016, SE = 0.003, *P* < 0.001) between the pre- and post-training visit. No significant change in mean diffusivity (MD; est = −0.008, SE = 0.004, *P* = 0.06) or isotropic volume fraction (ISOVF; est = 0.00, SE = 0.002, *P* = 0.83) was found.

Consistent with prior reports, we found all diffusion metrics to vary significantly along the dLGN-hMT+ tract. This was true both at each timepoint examined, and between timepoints (i.e. representing a potential effect of the training intervention, Fig. 5). The significant overlap between dLGN-V1 and dLGN-hMT+ tracts (Fig. 5) may obscure changes in the dLGN-hMT+ tract due to rehabilitation. As can be seen in Fig. 5, there was an increase in FA specifically in the distal, non-overlapping portion of the dLGN-hMT+ tract between the pre- and post-training timepoints (visits 2–3). Linear mixed model analyses were used to investigate differences in tract profiles in this distal portion of the dLGN-hMT+ tract across the two timepoints (model: diffusion_measure ∼ timepoint + node + (1|participant); Fig. 5). There was a significant increase in FA (est = −0.019, SE = 0.003, *P* < 0.001) and NDI (est = 0.009, SE = 0.003, *P* = 0.001) and reduction in ODI (est = −0.016, SE = 0.003, *P* < 0.001) between the pre-training and post-training timepoints. There was no difference in MD (est = −0.008, SE = 0.004, *P* = 0.06) or ISOVF (est = 0.00, SE = 0.002, *P* = 0.83) between the pre-training and post-training timepoints. This indicates that FA, NDI and ODI significantly changed with visual training (see Fig. 5). These improvements were specific to the ipsilesional distal dLGN-hMT+ tract and did not occur in the contralesional (‘sighted’) hemisphere (see Supplementary Fig. 3).

After intervention, a linear regression analysis showed that the mean change in FA along the distal dLGN-hMT+ tract was not directly related to improvements in NDR thresholds (adjusted *R*^2^ = 0.01, *F*(1,14) = 1.2; *P* = 0.29) or area of improvement on the HVF (adjusted *R*^2^=−0.08, *F*(1,12) = 0.1; *P* = 0.80; see Supplementary Table 4 for additional white matter metrics).

**Figure 6:**
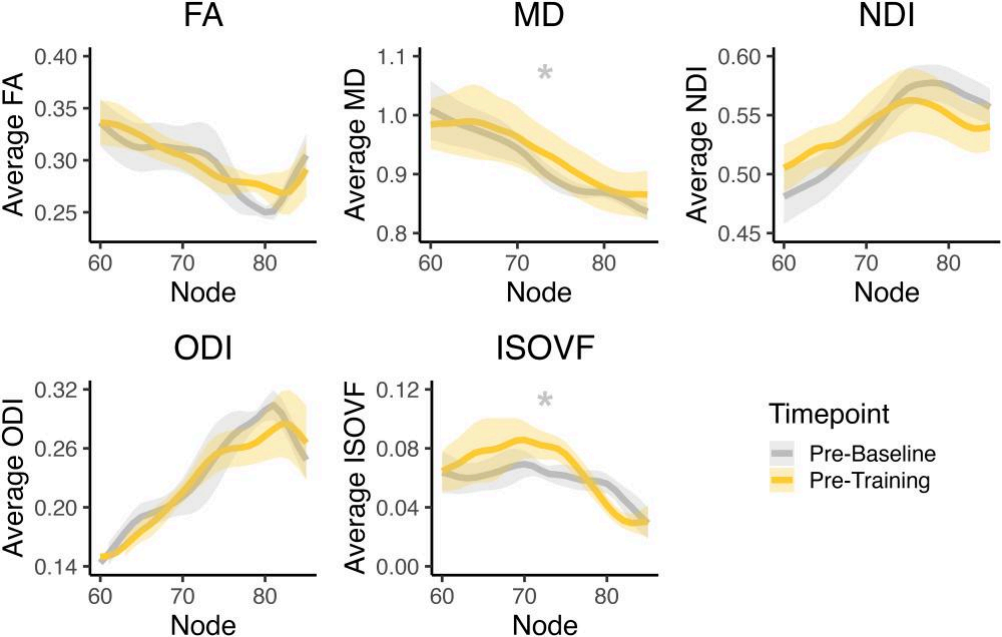
**The distal dLGN-hMT+ tract without training.** Diffusion metrics along the distal portion of the dLGN-hMT+ in the absence of training (i.e. between visits 1 and 2). Lines represent mean diffusion metrics across participants at each node, while shaded error bars reflect the SEM. Significant differences between timepoints as measured by a linear mixed-effects model are indicated by an asterisk (*). There was a significant increase in mean diffusivity (MD; est = −0.015, SE = 0.006, *P* = 0.008) and isotropic volume fraction (ISOVF; est = −0.007, SE = 0.002, *P* = 0.012) between the pre-baseline and pre-training visit. No significant change in fractional anisotropy (FA; est = −0.003, SE = 0.004, *P* = 0.53), neurite density index (NDI; est = −0.000, SE = 0.003, *P* = 0.92) or orientation dispersion index (ODI; est = 0.005, SE = 0.005, *P* = 0.28) were found.

To ensure that differences were not due to random changes in white matter over time, a comparable analysis was performed between visits 1 and 2 for eight participants (R004 was removed due to tracking issues between dLGN-hMT+). There was no evidence that FA (est = −0.003, SE = 0.004, *P* = 0.53), NDI (est = −0.000, SE = 0.003, *P* = 0.92) or ODI (est = 0.005, SE = 0.005, *P* = 0.28) changed over this time period (Fig. 6), suggesting no change without a training intervention. Of note, there was a significant increase in MD (est = −0.015, SE = 0.006, *P* = 0.008) and ISOVF (est = −0.007, SE = 0.002, *P* = 0.012), potentially reflecting ongoing degeneration in the absence of intervention.

#### Control analyses

Data quality control analyses indicated that there was no evidence that data quality varied across any of the three timepoints (see supplementary materials for details).

## Discussion

The present study investigated whether diffusion-weighted imaging measures of white matter microstructure are related to the performance outcome of visual rehabilitation post-stroke. After replicating prior work that reported improvements in visual motion discrimination and integration following training,^8,9^ we found no significant relationship between mean change in diffusion-weighted imaging measures across the entire length of any of the tracts analysed and visual performance improvements. However, we noted a significant increase in FA and NDI, and a reduction in ODI in the distal portion of the dLGN-hMT+ tract after the training intervention, but not between two untrained timepoints.

###  

#### Visual training alters microstructure in the distal, ipsilesional dLGN-hMT+ pathway in visual field deficits

In the motor system, training increases FA in motor tracts in both healthy participants^67^ and stroke survivors.^18^ In occipital stroke survivors, residual visual perceptual abilities in the blind field have been linked to plasticity in hMT+^68^ and the integrity of the distal portion of white matter pathway between the dLGN and hMT+ .^23^ Here, we asked if this tract, as well as the dLGN-V1 pathway are further impacted by visual training previously shown to recover global motion discrimination and integration at trained, blind-field locations.^8,9,16^ There was no significant relationship between the magnitude of change in FA across the entire dLGN-hMT+ or dLGN-V1 tracts and improvements in the trained motion discrimination task, or clinical HVF test. This is likely due to the significant overlap between the dLGN-V1 and dLGN-hMT+ pathways, discussed by Ajina *et al.*,^23^ that obscured training-related changes in the dLGN-hMT+ pathway. This was confirmed by an exploratory analysis of the distal, non-overlapping portion of the dLGN-hMT+ tract, which revealed a significant increase in FA after training, although the magnitude of change was not directly related to improvements in the tasks. There was also an increase in NDI and a decrease in ODI in the distal dLGN-hMT+ tract. This increase was not found in sighted dLGN-hMT+ (see supplementary materials), suggesting changes were specific to the trained hemisphere.

Increased FA after training reflects an increase in anisotropic, hindered water diffusion in the distal dLGN-hMT+ tract. These changes might represent increased white matter integrity of this pathway after training, with possible substrates including training-induced increases in myelination.^69^ The associated increase in NDI and decrease in ODI further indicate that these changes may involve an increase in the density of neurites and greater fibre orientation coherence in this pathway after training.^54^ Increased NDI and reduced ODI are known to be related to increased axonal growth and myelination,^70,71^ and therefore, these changes may underlie visual perceptual improvements from training. In contrast, there was no change in FA, NDI or ODI between two timepoints without training (visits 1–2). Thus, after 6-months of visual discrimination training in the blind field, chronic stroke survivors show notable changes in FA, NDI and ODI in the distal dLGN-hMT+ tract. Interestingly, neither MD nor ISOVF (a measure of extra-cellular water diffusion) showed any change with training, while both increased in the absence of training, between visits 1 and 2. Since increases in these measures can reflect a decrease in tissue density, this suggests ongoing retrograde degeneration of tracts over time, which is evident without training, and is potentially annulled by the 6 months of training.^72^

#### Limitations and future directions

##### Variability between timepoints

The current longitudinal study required scanning at three timepoints with extensive behavioural training between visits 2 and 3. For most participants, scanning sessions were roughly 6 months apart (days of training: median = 155, range = 102–271); however, some participants trained for considerably longer (in the case of R017, it was almost a year: 271 days of training). Added to this, there are several sources of variability in diffusion-weighted imaging, including noise, movement, participant alignment, partial volume effects and changes in MRI scanner characteristics. It is therefore possible that the variability of these sources at different timepoints impacted current results. Despite these concerns, previous studies have shown high reproducibility for diffusion-weighted measures of FA and MD,^73-75^ although they compared participants scanned within short scan intervals (usually days) and therefore might have underestimated the sources of variability relative to a longitudinal study like ours. Studies using longer time frames explored the test-retest reliability of DTI after one year^76,77^ and NODDI models after 4 weeks^78^ and found good evidence for the reproducibility of diffusion-weighted metrics. Thus, it is likely that the current study would show similar levels of reproducibility. Control analyses comparing (i) mean FA in the brain, (ii) mean FA of tracts of interest and (iii) signal to noise of the corpus callosum between the three timepoints suggest that data quality was similar across scanning sessions (see supplementary materials for details). These results further suggest that, without intervention, diffusion measures are stable. Additionally, it is possible that only measuring diffusion-weighted imaging parameters before and after the training might have also limited our ability to detect microstructural changes during training. Microstructural changes may change dynamically during learning over shorter time periods^79^ and therefore the pattern of changes (rather than the end result) might better explain why some individuals may be more likely to benefit from rehabilitation. A future study could investigate white matter markers at multiple timepoints throughout training to better understand the effects and dynamics of learning.

A major challenge in longitudinal patient-related research is the small sample size and dropout throughout the study. Twenty-four stroke survivors were recruited to the study, however only 20 of these completed 6 months of visual training. Those who dropped out did so due to the significant time commitment of the project. Moreover, of the 20 participants who completed rehabilitation, two could not be scanned (R016 and R021), and a further two (R004 and R006) had large occipital lesions that interfered with diffusion tractography within the visual cortex. In addition, visual field data for R002 and R014 were removed from analyses due to fixation losses outside of the normal range at one timepoint. This meant that for some of the analyses the sample was reduced to *N* = 14. Even with this level of dropout, the sample size is at least as large as previous studies investigating visual rehabilitation in chronic stroke^8,80,81^ and it is clear that larger studies are needed to investigate changes in diffusion after visual training. Additionally, in a larger sample size, it would be possible to run whole-brain analyses, such as Tract-Based Spatial Statistics, to determine whether there are more global changes in diffusion metrics with rehabilitation.

##### Future directions

The change in microstructural integrity of the dLGN-hMT+ pathway as a result of visual training suggests it is likely to be critical to the training-induced perceptual improvements reported by these patients. Future work should therefore focus on targeting these pathways to enhance rehabilitation efficacy and efficiency. Non-invasive brain stimulation has been used to enhance motor learning in healthy controls^82,83^ and stroke survivors^84-86^. In the visual system, non-invasive brain stimulation can enhance vision in healthy controls^87-89^ and has been proposed to improve vision loss from a range of disorders,^90^ including after V1 damage.^91,92^ The current research suggests that future studies should aim to target the dLGN-hMT+ pathway to enhance the effects of visual rehabilitation to restore conscious visual perceptual abilities.

## Conclusion

Six months of visual discrimination training in the blind field improves vision for both trained and untrained tasks. Diffusion-weighted tractography on open-access platform *brainlife.io* showed a change in microstructural properties in the distal section of the dLGN-hMT+ tract. These results add to the body of work suggesting an important role for the dLGN-hMT+ pathway in preserved visual processing abilities post V1 stroke, which can be recruited by training to restore conscious visual discrimination abilities in previously blind fields.

## Supplementary Material

fcae323_Supplementary_Data

## Data Availability

We confirm that upon in-principle acceptance, we registered the approved protocol on the Open Science Framework. The pre-registered Stage 1 manuscript is available here: https://osf.io/p6vcx/. We have also made openly available all appropriately anonymized raw data (https://osf.io/36eug/) supporting the reported analyses and analysis code (https://github.com/hanna-willis/rehab_diffusion) from this study. The data were published publicly at the end of the project to allow other researchers to reproduce our results and reuse our processing pipeline for future studies as described by Avesani *et al*.^39^
