# Supplementary material for: Rehabilitating homonymous visual field deficits: white matter markers of recovery—stage 2 registered report
Source: Brain Commun. 2024 Sep 23;6(5):fcae323. doi: 10.1093/braincomms/fcae323 (PMC11487913; doi:10.1093/braincomms/fcae323)
Supplement: fcae323_Supplementary_Data [file fcae323_supplementary_data.pdf]

Supplementary materials

Participants

Demographics

Supplementary Table 1. All participant demographics.

Participant	Stroke Type	Age Range (yrs)	Months Since Lesion	Deficit Side	Visits
R001	unknown	50-59	68	left	1 & 2
R002	unknown	20-29	39	right	1, 2 & 3
R003	ischaemic	30-39	58	both	1, 2 & 3
R004	haemorrhagic	50-59	31	left	1, 2 & 3
R005	haemorrhagic	60-69	21	left	1, 2 & 3
R006	ischaemic	20-29	13	left	2 &3
R007	haemorrhagic	40-49	7	left	2 &3
R008	unknown	40-49	32	left	1 & 2
R009	haemorrhagic	30-39	25	Left	1
R010	ischaemic	60-69	47	right	1, 2 & 3
R011	unknown	60-69	7	left	2 & 3
R012	unknown	70-79	58	left	1, 2 & 3
R013	haemorrhagic	70-79	26	right	2 &3
R014	ischaemic	30-39	30	right	2 &3
R015	ischaemic	30-39	29	right	1, 2 & 3
R016	haemorrhagic	60-69	40	right	2 &3
R017	ischaemic	60-69	6	right	2 &3
R018	ischaemic	30-39	26	right	2 &3
R019	ischaemic	40-49	31	left	2 &3
R020	unknown	40-49	297	left	2 &3
R021	unknown	50-59	11	left	2 &3
R022	ischaemic	40-49	14	right	2 &3
R023	unknown	60-69	49	right	2 &3
R024	ischaemic	60-69	13	left	2 &3

Training locations

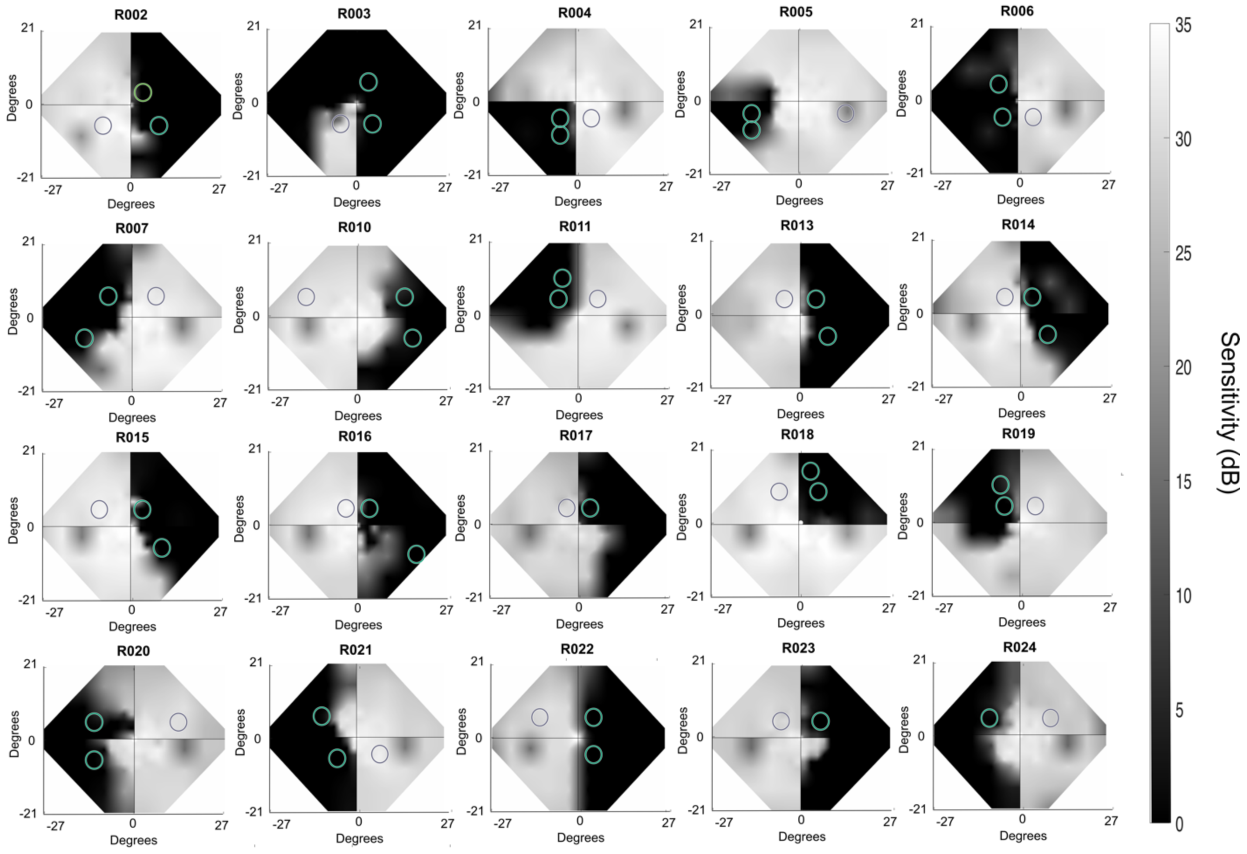

Supplementary Figure 1. Binocular composite Humphrey Visual Fields for all participants. The two blind field training locations are indicated in green and a matched sighted field location in grey. Three participants (R017, R023 and R024) only trained at one location in the blind field due to time constraints.

Fields for untrained participants

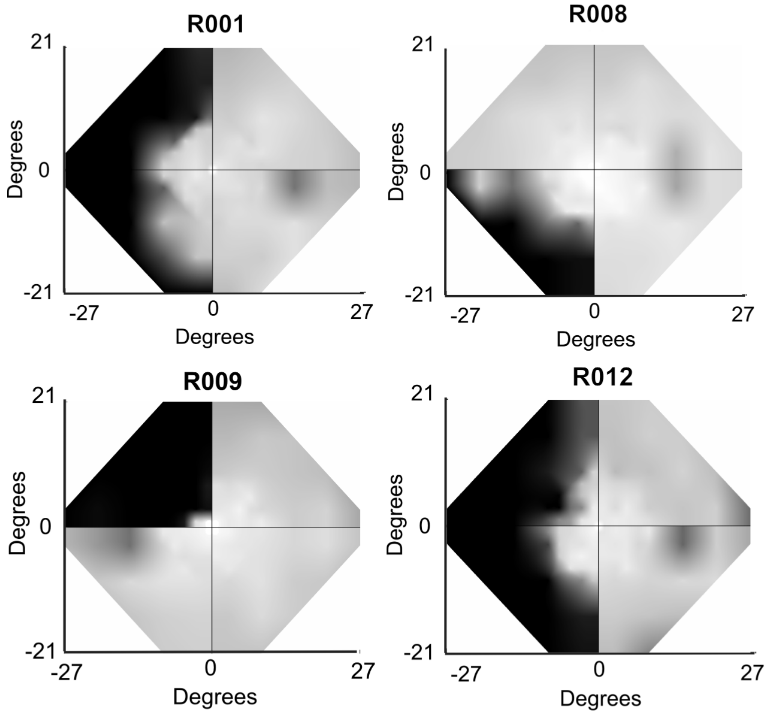

Supplementary Figure 2. Binocular composite Humphrey Visual Fields for untrained participants. Four participants R001, R008, R009 and R012 did not complete training.

Power Analysis

To the best of our knowledge, there are no previous publications from which we can estimate effect sizes for white matter microstructural changes. We therefore computed a sensitivity analysis to determine the effect sizes to which we would be sensitive with an achievable sample size. Due to the commitments of the study and rarity of isolated visual field deficits, a sample size of 20 was deemed appropriate. We used linear regression to determine the relationship between visual improvement and FA change in this study.

The sensitivity power analysis was calculated for a linear regression analysis using the 'pwr' package in R (R Core Team, 2020) with a sample of size 20, alpha of 0.05, and power of 0.9. From this calculation, we are powered sufficiently to detect large effect sizes (Cohens F = 0.59)

90% of the time. We used a one-directional test, as we predicted that both behavioural measures and FA would increase with training. In our view, a moderate-to-large effect size is necessary to have the impact on activities of daily living expected from the training particularly given the duration of the planned intervention. Smaller effect sizes will likely have less real-life significance.

Although there are no known studies investigating the relationship between FA change and visual improvement in visual field defects on which to base effect sizes, we were confident that large effect sizes would be achievable in this study. Behavioural training studies in visual field defects found large or very large effect sizes for within and between-subject improvements in motion discrimination thresholds (n=17 within-subject d=2.78; n=5 untrained, 17 trained; between-subject d=4.49; (Cavanaugh and Huxlin, 2017) and on Humphrey Visual Field measurements (Cavanaugh and Huxlin, 2017; Halbertsma *et al.*, 2020).

Additionally, studies in the motor cortex report large effect sizes showing significant increases in FA in motor skill training after a 6-week intervention (n=48; partial $eta^2 = 0.17$; (Scholz *et al.*, 2009). Moreover, Fan et al (2015) reported a large effect showing that increased FA in ipsilesional motor tracts in stroke survivors was related to improvements in motor function after a 4-week intervention (n = 10; r = 0.68; (Fan *et al.*, 2015). Based on this literature, we therefore predicted that we would be sensitive to finding a similarly large effect size in a 6-month intervention in twenty stroke survivors. Thus, according to our sensitivity analysis we were well powered (90% power) to investigate the relationship between improvements in vision and change in FA.

Methods

Pipeline apps

Supplementary Table 2. Our pipeline consisted of 15 *brainlife.io* Apps. Description, weblinks and DOI for the open cloud app services used to process these data.

	Application	Github Repository	Open Service DOI
**Anatomical pre-processing**	FSL Anat	*https://github.com/brainlife/app-fsl-anat*	10.25663/brainlife.app.273
	Freesurfer	https://github.com/brainlife/app-freesurfer	10.25663/bl.app.0
	Multi-atlas tool	https://github.com/faskowit/app-multiAtlasTT	10.25663/brainlife.app.470
	Tissue-type segmentation	https://github.com/brainlife/app-mrtrix3-5tt	10.25663/brainlife.app.239
**Diffusion pre-processing**	mrtrix3 preprocess	*https://github.com/brain-life/app-mrtrix3-preproc*	10.25663/bl.app.68
	Brain extraction of DWI	https://github.com/brainlife/app-FSLBET	10.25663/brainlife.app.163
**Regions of interest**	Apply warp from subject-to-standard space to ROIs (T1w)	https://github.com/brainlife/app-register-rois-mni	10.25663/brainlife.app.715
	Reslice ROIs to DWI space	https://github.com/bacaron/app-reslice-rois-dwi	10.25663/brainlife.app.671
	Generate all ROIs from Glasser parcellation	*https://github.com/brainlife/app-roiGenerator*	10.25663/brainlife.app.592
	Generate visual ROIs from Glasser parcellation	https://github.com/brainlife/app-roiGenerator	10.25663/brainlife.app.411
	Combine ROI	https://github.com/svincibo/app-combineROI	10.25663/brainlife.app.313
	FSLmaths	https://github.com/hanna-willis/app-roi-fslmaths	10.25663/brainlife.app.680
**Diffusion processing**	FSL DTIFIT	*https://github.com/brainlife/app-fslDTIFIT*	10.25663/brainlife.app.292
	NODDI	*https://github.com/brainlife/app-noddi-amico*	10.25663/brainlife.app.365
**Tractography**	Trekker ROI Tracking (DWI)	*https://github.com/brainlife/app-trekker-roi-tracking*	10.25663/brainlife.app.355
	Remove Tract Outliers	*https://github.com/brainlife/app-removeTractOutliers*	10.25663/brainlife.app.195
	Tract Analysis Profiles	https://github.com/brainlife/app-tractanalysisprofiles	10.25663/brainlife.app.361

Additional analyses

Average FA across timepoints

Average FA for each tract of interest was calculated across timepoints. As can be seen in Supplementary Table 3, there was limited evidence of variation between timepoints.

Supplementary Table 3. Average measures of FA in all tracts of interest

Tract of Interest	Diffusion Metric	Timepoint	Median (IQR)
VPL-S1	FA	Pre-baseline	0.38 (0.03)
		Pre-Training	0.35 (0.03)
		Post-Training	0.36 (0.19)
dLGN-V1	FA	Pre-baseline	0.35 (0.02)
		Pre-Training	0.37 (0.04)
		Post-Training	0.37 (0.05)
dLGN-hMT+	FA	Pre-baseline	0.35 (0.04)
		Pre-Training	0.35 (0.35)
		Post-Training	0.35 (0.36)

Changes in the distal dLGN-hMT+ tract

Relationship to behaviour

Linear regression analyses were used to study the relationship between behaviour and white matter metrics in the distal dLGN-hMT+ tract. No significant relationships were found between any diffusion measures in the distal dLGN-hMT+ tract and improvements in NDR thresholds or area of improvement on the HVF (see Supplementary Table 4). These results indicate that although there is an increase in FA and NDI and reduction in ODI after six months of visual rehabilitation, these changes were not related to the magnitude of improvements in NDR thresholds or area of improvement.

Supplementary Table 4. *Linear regression analyses of change in NDR threshold or area of improvement on Humphrey Visual Fields (HVF) and additional diffusion metrics for the distal dLGN-hMT+ tract.*

Task	Diffusion measure	Adjusted R^2	F Statistic	Degrees of Freedom	P Value
NDR Threshold Change	FA	0.01	1.2	14	0.29
	MD	0.05	1.8	14	0.20
	RD	0.12	3.0	14	0.11
	AD	-0.05	0.3	14	0.58
	NDI	0.03	1.5	14	0.24
	ODI	-0.06	0.2	14	0.65
	ISOVF	0.06	1.9	14	0.19
Area Improved on HVF	FA	-0.08	0.1	12	0.80
	MD	-0.05	0.3	12	0.57
	RD	-0.07	0.2	12	0.69
	AD	-0.05	0.4	12	0.53
	NDI	-0.08	0.0	12	0.91
	ODI	-0.08	0.0	12	0.90
	ISOVF	0.08	2.1	12	0.17

Changes in the sighted hemisphere

To further explore whether changes in the blind distal dLGN-hMT+ were specific to training, we also investigated changes in diffusion metrics in the sighted distal dLGN-hMT+. An additional linear mixed effects model was used with hemisphere as an additional fixed factor. (model: diffusion_measure ~ hemisphere * (timepoint + node) + (1|participant)).

As can be seen in Supplementary Figure 3, there was a significant increase in FA in the blind field (est=-0.019, SE=0.003, Holm-Bonferroni corrected p=0.001) but not the sighted hemisphere (est=0.019, SE=0.003, Holm-Bonferroni corrected p=0.100). There was no change in MD in the blind hemisphere (est=0.008, SE=0.004, Holm-Bonferroni corrected p=0.720) but a significant increase in MD in the sighted field (est=-0.017, SE=0.004, Holm-Bonferroni corrected p=0.003). There was a significant increase in NDI in the blind hemisphere (est=-

0.009, SE=0.003, Holm-Bonferroni corrected p=0.049) and decrease in the sighted hemisphere (est=0.009, SE=0.003, Holm-Bonferroni corrected p=0.049). There was a significant decrease in ODI in the blind hemisphere (est=0.016, SE=0.004, Holm-Bonferroni corrected p=0.001) and no change in the sighted field (est=-0.005, SE=0.004, Holm-Bonferroni corrected p=1.000). Finally, there were no changes in ISOVF in either hemisphere (blind: est=-0.0003, SE=0.002, Holm-Bonferroni corrected p=1.000; sighted: est=-0.005, SE=0.002, Holm-Bonferroni corrected p=0.172).

The significant increase in MD and decrease in NDI in the sighted hemisphere might indicate slight degeneration of this tract. Human ageing is known to be associated with progressive changes in white matter architecture and this includes demyelination, loss of cortical dendritic spines and axonal alterations (Pannese, 2011; Pakkenberg et al, 2003). Moreover, diffusion imaging studies have found reduced FA and increased MD in older adults (Burzynska et al; Vernooij et al 2008; Pferrerbaum & Sullivan, 2003). The age of the current population ranged between 24 and 71 years old at the pre-training visit. Post-training scans were then collected between six months and one year later. It is possible that ongoing ageing between the pre- and post-training visits could explain the degeneration in this tract. Moreover, this might suggest that the blind hemisphere would show similar levels of degeneration, so positive effects of training may have prevented this change.

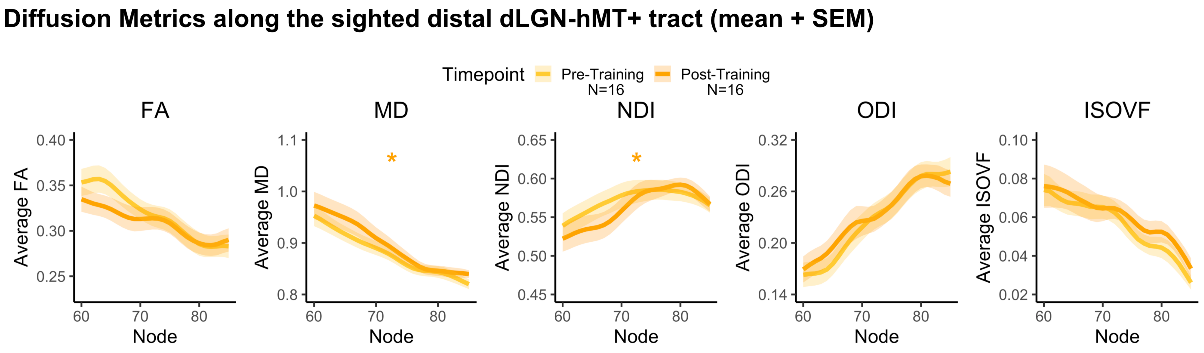

Supplementary Figure 3. The sighted distal dLGN-hMT+ tract. *Diffusion measures along the distal portion of the dLGN-hMT+ in the sighted hemisphere. Lines represent the mean diffusion metric across participants at each node, while shaded error bars reflect the standard error of the mean. Significant differences between Visits 2 and 3 (pre- to post-training) are indicated by an asterix (*). There was a significant increase in MD and decrease in NDI. There were no changes in FA, ODI or ISOVF.*

<u>Alternative pathways</u>

Alternative pathways have also been suggested to be involved in blindsight, such as the superior colliculus/pulvinar pathway to hMT+ and ipsilateral hMT+ to contralateral hMT+. The pathway between the superior colliculus and hMT+ likely relays via the inferior pulvinar (Lyon, Nassi and Callaway, 2010). It is therefore unclear whether this pathway can be distinguished (i.e. independently tracked using diffusion-weighted imaging; Ajina et al., 2015) from that between inferior pulvinar and hMT+. Due to this uncertainty, we decided that investigating these structures with diffusion-weighted imaging is outside of the scope of this current paper.

Data quality checks

To ensure consistency across repeated scans, the mean FA was calculated across the brain (brainlife.app.746) and compared between the pre-baseline (mean=0.26; sd=0.02), pre-training (mean=0.26; sd=0.02) and post-training (mean=0.26; sd=0.02) visits. A linear mixed effects model indicated that mean FA across the brain did not significantly differ between the pre-baseline and pre-training *(est=-0.004, SE=0.002, p=0.226)* or pre-training and post-training *(est=-0.002, SE= 0.002, p=0.595)* timepoints.

A second control analysis calculated the signal-to-noise ratio in the corpus callosum (brainlife.app.120) between the pre-baseline (mean=24.4; sd=8.77), pre-training (mean=25.4; sd=7.87) and post-training (mean=26.7; sd=6.96) visits. There was no significant difference in signal-to-noise ratio of the corpus collosum between the pre-baseline and pre-training *(est=0.88, SE=1.89, p=0.89)* or pre-training and post-training *(est=83, SE=1.42, p=0.83)* timepoints.

Finally, for specific tracts of interest, paired t-tests showed that in a subset of 9 participants there was no change in mean FA between pre-baseline and pre-training visits in the dLGN-hMT+ *(t(7)= 1.95, p=0.092*), dLGN-V1 *(t(8)=-0.97, p=0.363)* or VPL-S1 tract *(t(7)=0.94, p=0.379).* Based on these analyses, there is no evidence that the data quality varied between timepoints.

Supplementary References

Ajina, S. *et al.* (2015) 'Human blindsight is mediated by an intact geniculo-extrastriate pathway', *eLife*, 4. Available at: https://doi.org/10.7554/eLife.08935.

Cavanaugh, M.R. and Huxlin, K.R. (2017) 'Visual discrimination training improves Humphrey perimetry in chronic cortically induced blindness', *Neurology*, 88(19), pp. 1856–1864. Available at: https://doi.org/10.1212/WNL.0000000000003921.

Fan, Y.T. *et al.* (2015) 'Changes in structural integrity are correlated with motor and functional recovery after post-stroke rehabilitation', *Restorative Neurology and Neuroscience*, 33(6), pp. 835–844. Available at: https://doi.org/10.3233/RNN-150523.

Halbertsma, H.N. *et al.* (2020) 'Functional connectivity of the Precuneus reflects effectiveness of visual restitution training in chronic hemianopia', *NeuroImage: Clinical*, 27. Available at: https://doi.org/10.1016/j.nicl.2020.102292.

Lyon, D.C., Nassi, J.J. and Callaway, E.M. (2010) 'A Disynaptic Relay from Superior Colliculus to Dorsal Stream Visual Cortex in Macaque Monkey', *Neuron*, 65(2), pp. 270–279. Available at: https://doi.org/10.1016/j.neuron.2010.01.003.

R Core Team, . (2020) 'R: A language and environment for statistical computing. R Foundation for Statistical Computing, Vienna, Austria'.

Scholz, J. *et al.* (2009) 'Training induces changes in white-matter architecture', *Nature Neuroscience*, 12(11), pp. 1370–1371. Available at: https://doi.org/10.1038/nn.2412.
